# The Microfluidic Technique and the Manufacturing of Polysaccharide Nanoparticles

**DOI:** 10.3390/pharmaceutics10040267

**Published:** 2018-12-09

**Authors:** Enrica Chiesa, Rossella Dorati, Silvia Pisani, Bice Conti, Gloria Bergamini, Tiziana Modena, Ida Genta

**Affiliations:** Department of Drug Sciences, University of Pavia, Viale Taramelli 12, 27100 Pavia, Italy; enrica.chiesa01@universitadipavia.it (E.C.); silvia.pisani01@universitadipavia.it (S.P.); bice.conti@unipv.it (B.C.); gloria.bergamini01@universitadipavia.it (G.B.); tiziana.modena@unipv.it (T.M.); ida.genta@unipv.it (I.G.)

**Keywords:** microfluidic, nanoparticle production, polysaccharide nanoparticles, hyaluronic acid, chitosan, alginate

## Abstract

The microfluidic technique has emerged as a promising tool to accelerate the clinical translation of nanoparticles, and its application affects several aspects, such as the production of nanoparticles and the in vitro characterization in the microenvironment, mimicking in vivo conditions. This review covers the general aspects of the microfluidic technique and its application in several fields, such as the synthesis, recovering, and samples analysis of nanoparticles, and in vitro characterization and their in vivo application. Among these, advantages in the production of polymeric nanoparticles in a well-controlled, reproducible, and high-throughput manner have been highlighted, and detailed descriptions of microfluidic devices broadly used for the synthesis of polysaccharide nanoparticles have been provided. These nanoparticulate systems have drawn attention as drug delivery vehicles over many years; nevertheless, their synthesis using the microfluidic technique is still largely unexplored. This review deals with the use of the microfluidic technique for the synthesis of polysaccharide nanoparticles; evaluating features of the most studied polysaccharide drug carriers, such as chitosan, hyaluronic acid, and alginate polymers. The critical assessment of the most recent research published in literature allows us to assume that microfluidics will play an important role in the discovery and clinical translation of nanoplatforms.

## 1. Introduction

Microfluidics is the science and technology field dealing with miniaturized systems that process, or manipulate, small volumes of fluids (10^−9^ to 10^−18^ L), by means of channels with dimensions ranging from tens to hundreds of micrometers. An interesting paper published in Nature in 2006 highlighted how this, by that time, emerging technique could be profitable in several areas, such as molecular biology, molecular analysis, biodefence, and microelectronics [1]. Twelve years have passed and microfluidics is successfully applied in many different areas. 

The basic principles of the Microfluidic technique reside in the mechanisms and laws governing mixing in a narrow environment. They have been investigated and reported by several authors along these years, as the few examples cited herewith [2,3,4,5,6,7,8,9,10]. A recent paper of Capretto and coll. [11] reviews the technique, pointing out its principles. The main feature of this technique is the ability to translate old bulk techniques into a microchannel with a width of about 100 μm. In this channel, chemicals are mixed using a pumping technique for synthesis and separation analysis purposes. Talking about this technique, it should be said that microfluidic mixing is not governed by the same laws applied at the macroscale and, further, microfluidic devices are not the miniaturization of macroscale ones. Physical characteristics and diffusion-based mass transfer cannot be scalable linearly from macroscale to microscale. The main feature of the microfluidic technique is the laminar flow that cannot be reached in macroscale devices. This phenomenon is due to the predominant role of viscous forces and it cannot be neglected. In other words, a microfluidic mixer is not simply a miniaturized version of a macroscale mixing device, and it should be designed in such a way to leverage the physical characteristics of mass and fluid transfer in the micro-confined domain.

Briefly, fluid flow can be described by two regimens: Turbulent and laminar. The formation of a flow vortex occurs in the turbulent regimen, meanwhile fluid runs in parallel layers in the laminar flow condition and there is no current perpendicular to flow direction. Different inertial and viscous forces characterize these two differential regimens that can be revealed by Reynolds number (Re) as defined by Equation (1):

Re = ρuD/μ = uD/v(1)

where ρ and μ are the fluid density and dynamic viscosity respectively; v is the fluid viscosity; u is the mean fluid velocity, and D is the hydraulic diameter of the channel. D depends on the cross-sectional geometry of the channel, and is given by Equation (2):

D = 4A/P(2)

where A is the channel cross-sectional area and P is the channel wet perimeter.

High Re (typically Re > 100) is related to turbulent flows, and it is dominated by random motion, in which there are advective mass transport phenomena in all spatial directions [12]. Low Re (Re < 100) is associated to laminar flow, where viscous forces overcome inertial effects, and mixing between streams depends on the molecular diffusion. A transitional flow regimen usually occurs between laminar and turbulent regimens. The exact transitional Reynolds number is a function of many parameters, including channel geometry and channel surface roughness. In a microfluidic system, Re values are lower than 100, corresponding to laminar flow, so flow mixing is dominated by passive molecular diffusion in microfluidic devices. Diffusion is the molecular transport from a higher concentration area to a lower concentration region.

The diffusion process can be explained by Fick’s laws (3):

J = −D × dφ/dx(3)

where φ is the concentration of a chemical substance, x is a spatial coordinate, and D is the diffusion coefficient. Using spherical particles, D can be calculated by Stokes–Einstein Equation (4):

D = kT/6πμr(4)

where k is the Boltzmann constant, T is absolute temperature, μ is the fluid flow dynamic viscosity, and r is the particle radius. 

The required time (t) for species diffusion is in quadratic correlation with the distance covered (x). This concept is described by the following Equation (5):

x^−2^ = 2D × t (5)

where x is the distance covered in time (t), and D is the diffusion coefficient. On a microscale, the diffusion distance can be extremely small, especially if fluid streams are hydro-dynamically focused. Because time depends on the square power of x, a decrease in the diffusion distance has the effect of dramatically reducing the time required for complete mixing to be achieved.

Microfluidic platforms are often made of monocrystalline silicon thanks to its physicochemical characteristics, but glass borosilicate is also a good material for microfluidic devices. In the latest years, polymers were used to produce microfluidic apparatuses. In particular, poly(dimethylsiloxane) (PDMS) has become the favorite material for fabrication of microfluidic devices. It can be easily molded and patterned into channels, it can reproduce micrometer-sized features with high fidelity, it is optically transparent, and it has low permeability to water. PDMS is; however, not resistant to organic solvents, like amines, strong acids, and hydrocarbons. This is the main disadvantage of PDMS, so solvent-resistant microfluidic reactors are being developed [13,14].

As explained above, mixing induced by laminar flow is one of the most important characteristics improving the performances of micro-scale devices. The micromixers can be classified into active and passive mixers.

Active mixers are provided with external sources that physically agitate the liquid in a microchannel. They can be acoustic or ultrasonic waves with acoustic actuators, magnetic disturbances with magnetic particles and an external magnetic field, pressure disturbance with the repeated stopping and flowing of the fluids, etc. [15,16]. On the other hand, passive mixers involve the hydrodynamic manipulation of the fluids. For example, chaotic advection, enhanced molecular diffusion, utilization of surface tension, fluid lamination, sequential splitting, and the combining of fluids inside the microchannel are typically of passive mixers. Mixers based on passive chaotic advection are the most commonly used; however, because of their simple design and fabrication, in addition to their higher mixing efficiency. Chaotic micromixers consist of geometries embedded into the microchannel to disturb the laminar flow. One of the most efficient chaotic micromixers is the Staggered Herringbone Mixer (SHM), originally developed by Stroock [4].

Kwak and coll. [15] describe the SHM in detail, also referring to previous studies looking at how to optimize the micromixer geometry. Briefly, the SHM consists of repeated patterns of grooves on the bottom of the microchannel. The individual groove is composed of two different length channels connected to each other, one relatively longer and the other relatively shorter, and those two grooves meet with a certain angle, usually 45 degrees. Studies on SHM geometry outlined that mixing is more affected by the depth of the groove than the angle, and numerical simulations verified the highest mixing efficiency to be in the range 0 < Re < 100 [4,17].

In the pharmaceutical area, the microfluidic technique can be used for different applications: synthesis, separation and sample analysis, organ-on-chip creation, and the production of nanoparticles (NPs). The last implementation involves the synthesis of nanoparticulate drug delivery systems, such as polymer NPs, liposomes, and solid lipid NPs (SLNs). This review will briefly describe the applications of the microfluidic technique in sample analysis and organ-on-chip creation, and it will focus on the use of the microfluidic technique for the production of polymer NPs, namely polysaccharide NPs.

## 2. Microfluidic Technique Applications

### 2.1. Synthesis, Separation, and Sample Analysis

Rapid and efficient diagnosis is an important way to improve human life expectancy. Analyses of biomarkers, the biomolecular indicators of medical conditions, hold excellent potential for the clinical diagnosis of various diseases. These biomarkers are frequently found in complex biological matrices or body fluids, and often they require sample preparation prior to analysis. Sample preparation steps often need large volumes (>mL) which further increase the analysis time and cost. Thus, fast and effective sample preparation techniques are needed to facilitate early diagnosis [18].

The microfluidic technique can aid the achievement of this aim, thanks to its rapid analysis and the small size of the sample required [19]. For this reason, biomarker analysis has been one of the most actively pursued applications of the miniaturization of chemical analyzers. 

Recently, different microfluidic devices have been developed to perform the sample preparation steps: Purification, sample preparation before analysis, separation, and detection [20]. On-chip sample preparation can be used to selectively extract, preconcentrate, and label target analytes in an automated way. For example, antibodies or aptamers are fixed on a solid support in order to purify complex samples, such as blood, from undesired matrix samples. These compounds have a high affinity to their target molecules and permit the selective capture of the desired molecules from a complex sample matrix, such as blood [21].

Moreover, analyte labeling is a fundamental sample preparation step that can be performed on a chip. On-chip labeling requires loading, reacting, and purifying, and typically uses a solid support inside the microchannels; fluorescent labeling is the most common approach being explored.

The application of microfluidic to capillary electrophoresis for protein separation and analysis seems to be a topic of great interest, and it has been recently experimented and reviewed [22]. Different examples of analysis-on-chip are reported in literature. Herzog and coll. [23] developed an integrated microfluidic device on a glass substrate for electrokinetic labeling and the separation of peptides and proteins. Shameli and coll. [24,25] developed a hybrid PDMS–glass microfluidic chip for the two-dimensional separation of proteins. The authors combined micellar affinity gradient focusing (MAGF) with the temperature gradient. Separation performances were tested on diverse compounds, such as Alexa Fluor dyes, fluorescent dyes, and fluorescently labeled pI markers. Sodium dodecyl sulfate-polyacrylamide and poly-SUS surfactants were used. The authors got good, promising results as they achieved a two-fold improvement in the peak capacity and separation resolution, if compared with the traditional method. 

### 2.2. Organ-On-Chip

Organs-on-chips are microscale devices, combining conventional cell culture methods with microfabrication and microfluidics technologies [26,27,28]. The term “organs-on-chips” was coined and it is used interchangeably to describe a single or multiple organ(s) on a chip. Microfluidics enables the precise regulation and control of fluids (cell culture media that act as blood substitutes and mimic the circulatory system), such that fluid turbulence is drastically reduced and the flow is laminar. One of the important consequences of laminar fluid flow is the generation of chemical gradients, which have an important bearing on cell migration and differentiation [29]. Circulating cell culture medium was used because gas exchange is a crucial factor in preserving cell physiology and function, a dissolved oxygen sensor was incorporated within this system to demonstrate oxygen adequacy in the culture chambers. 

Subsequently, researchers developed chips to study the behavior of various cell types in different pathologic situations. In example Song and coll. developed a microfluidic vascular chip to study adhesion of endothelial cells with circulating breast cancer cells; in this system endothelial cells simulated blood vessels [30]. Park and coll. fabricated a microfluidic device that permitted the culture of various types of neurons along with the separation of soma from axons in different compartments, thus, serving as a useful research tool to study nervous system injury and neurodegeneration [31]. 

Another promising approach is to use organs-on-chips for testing drug safety. As an example, Jang and coll. developed an effective microfluidic device that allowed the culture of renal tubules to test drug safety and efficacy [32]. The organ-on-chip was made by a polydimethyl siloxane (PDMS) microfluidic channel and a porous membrane substrate. In the study, the authors detailed how they set up the system with hydrodynamic conditions suitable to simulate optimal fluidic conditions for the cultured cells [28].

The final goal of developing organs-on-chips is the development of “human-on-a-chip” models that consist of interconnected compartments, each containing a cell type representing a different organ, linked through a microfluidic circulatory system [33]. Microengineered cell-culture systems that mimic complex organ physiology have the potential to be used for the development of human-relevant disease models, which are more predictive of drug efficacy and toxicity in patients. They can also provide greater insight into the drug mechanism of action and the accurate determination of drug pharmacokinetic. These systems can realistically help the pharmaceutical industry, which is under intense pressure economically, ethically, and scientifically, to find ways to accelerate the drug-development process, and to develop safer and more effective drugs at lower costs [26]. Due to the relatively new technique, organ-on-chip have a lack of regulation for their official use as drug evaluation systems. Currently, and to date, pharmacodynamic studies can exploit organ-on-chip potential because no models exist for these studies. The same does not occur for pharmacokinetic studies where specific animal models are required for the tests. From a regulatory stand point, organs-on-chips can be considered as analytical instruments for the purpose of drug testing. Therefore, they should comply with Good Laboratory Practices regulations in terms of calibration, validation, and qualification. However, not all regulations referring to analytical instruments can be applied to organs-on-chips, and regulation is still needed. 

### 2.3. Synthesis of Polymer Nanoparticles (NPs)

The microfluidic technique offers the opportunity to control the reaction environment in a very refined way. This is the main advantage of the application of the microfluidic technique in nanomedicine for drug delivery. The control of the reaction environment leads to improve the quality of nanoparticulate drug delivery systems (NPs, liposomes, solid lipid nanoparticles (SLN)), better modulating their size and drug loading, and eventually improving the preparation process yield of nanoparticles. Microfluidic reactors enable the rapid mixing of reagents, the control of temperature, and the precise spatio-temporal manipulation of reactions. All these factors are difficult, if not impossible, to be controlled in larger reactors [34,35]. In conventional, bulk synthesis methods, mixing is heterogeneous and typically occurs on a time scale longer than the characteristic time scale for self-assembly. For this reason, large and polydisperse nanosystems are obtained. On the contrary, the controlled and homogeneous mixing in microfluidic synthesis methods results in smaller and uniform nanosystems. In microfluidic synthesis of nanosystems, the physicochemical properties of nanosystems, determining their in vivo fate, can be precisely controlled in a reproducible manner [36,37].

As long as NPs are concerned, the traditional batch methods lack precise control over the mixing and supersaturation level, leading to uncontrolled nucleation and growth processes, resulting in poor control over final particle characteristics. In microfluidic devices, reactions are carried out within small reaction channels, with diameters between a few tens and a few hundreds of microliters. The small dimension enables homogeneous, fast (in the order of fraction to hundreds of milliseconds), and reproducible mixing conditions; heat and mass transfer can dramatically improve NPs process yield, drug loading, and size distribution while reducing the formation of undesirable by-products. These characteristics are fundamental because they, in turn, determine the physio-chemical properties of the produced nanomaterials [11,38]. 

Thus, the main process parameters are mixing time, channel size, and its geometry. These parameters are connected. Mixing time, τ_mix_, is recognized by different authors as a key parameter for the formation of NPs through precipitation, as it directly affects the dimensional properties of the produced nanomaterials [39,40]. A short mixing time can ensure a homogeneous spatial environment during the precipitation process, which in turn leads to uniform reaction conditions and particle formation kinetics. A short mixing time cannot be achieved in the bulk production of NPs. The mixing time can be shortened by studying the architecture of a mixing device. The architecture of microreactors plays a fundamental role in the fluidic domain and allows for the generation of a hydrodynamic-focusing flow pattern. Therefore, the mixing process is controlled by varying the width of the focused stream (w_f_), and provides a direct means to control the characteristics of NPs, as reported in the literature [41]. The theoretical mixing time for flow-focusing reactors (τ_mix_) was estimated according to Equation (6):

τ_mix_ ≈ (w_f_^2^)/4D (6)

where D represents the solvent diffusion coefficient and w_f_ the width of the focused stream. Following the simplified model represented by this equation, a greater diffusion coefficient corresponds to a shorter mixing time, and the smaller the width of the focused stream, the shorter the mixing time. Ideal behavior could be obtained by combining solvents characterized by high D values, with small w_f_ values, obtained by means of small microchannel sizes. However, this estimation represents only a largely simplified model, and many other factors should be considered to understand the effect of the microfluidic environment on the synthesis of nanomaterials.

**Figure 1:**
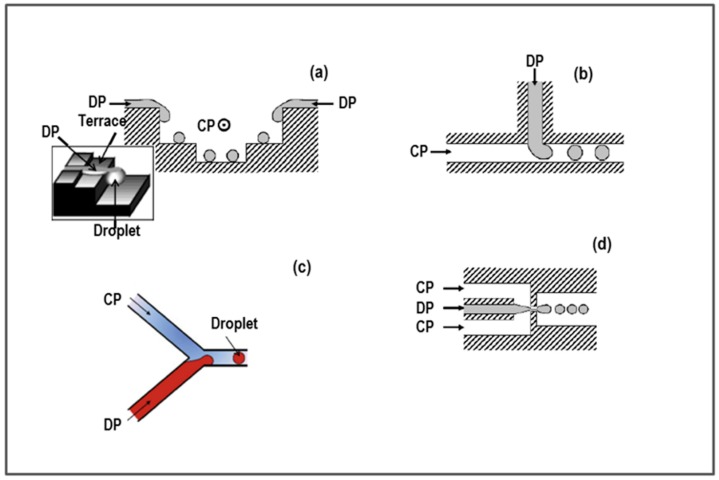
Microfluidic devices based on microchannel: (**a**) Terrace-like device; (**b**) T-junction device; (**c**) Y-junction device; and (**d**) flow-focusing microchannel device (FFD). CP and DP are the continuous and disperse phases, respectively.

In the different types of microfluidic devices, the developed channel geometry results in different droplet generation mechanisms. Four structures are commonly found in microchannel-based devices (Figure 1): Terrace-like microchannel, T-junction microchannel, Y-junction microchannel, and flow-focusing microchannel devices (FFD). In the terrace-like microchannel devices, several microchannels deliver the dispersed phase at the top and from both sides of the main channel, where the continuous phase flows (Figure 1a). The compressed dispersed phase passes onto to a flat surface (terrace), and the droplet forms as the dispersed phase thread falls from the flat surface in a deeper well. In T-junction microchannel devices (Figure 1b), the to-be-dispersed phase is delivered through a microchannel perpendicular to a main channel, in which the continuous phase flows. Thread breaks up and droplet formation takes place at the junction of the two microchannels, or further downstream. Droplets forms perpendicular to the main channel and are dragged out by parallel continuous phase flow. Jamalabadi et al. [42] investigated the effect of the injection angle, density ratio, and viscosity, on droplet formation. They concluded that injection angle close to perpendicular and parallel conditions worked to increase the diameter of droplets. In these conditions, the reduction of the diameter of droplets could be obtained by increasing flow rate, density ratio, and viscosity.

Y-junction microchannel devices represent a variation of the T-junction microchannel device. The disperse phase is delivered through a microchannel perpendicular to another channel, in which the continuous phase flows (see Figure 1c). These two channels merge to form the main channel. At first, the contact angle between the disperse phase and continuous phase is 90°, then the disperse phase undergoes about 120° incline, merging into the main channel. 

According to mathematical models shown by Jamalabadi and coll., these conditions should favor the formation of small sized droplets. 

FFDs (Figure 1d) are based on the principle of hydrodynamic focusing. The disperse phase flows in a central microchannel while the continuous phase is delivered through two side channels. Mixing of the continuous phase with the disperse phase happens at the restriction point, and at the same time the droplet is generated. Therefore, mixing takes place in a very narrow environment with controlled laminar flow conditions.

**Figure 2:**
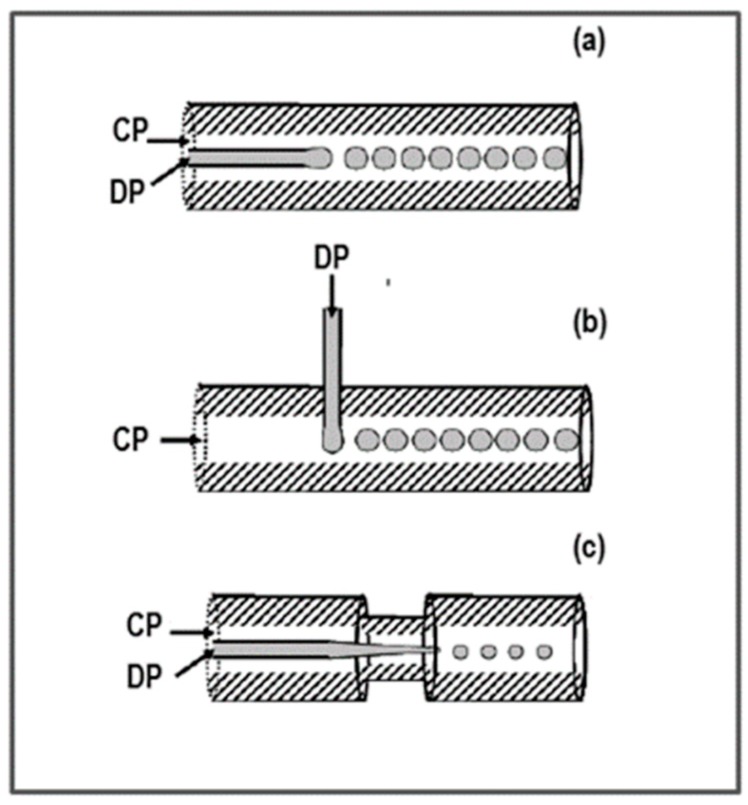
Capillary based devices: (**a**) Co-flow device; (**b**) cross-flow device; and (**c**) FFD device.

The same theoretic principle is exploited in three capillary-based devices. In this case the disperse phase flows in the very center line of the continuous phase flow (Figure 2), and it never meets the device wall. This is an advantage because the interaction of the disperse phase with the device material is minimized or cancelled. Three capillary-based devices are commonly found: Co-flow capillary device, cross-flow capillary device, and flow-focusing capillary device (Figure 2). In the co-flow device, the disperse phase flows in the same direction as the continuous phase (Figure 2a). The cross-flow device is based on the same principle as the T-junction microchannel device, where the disperse phase flows in a direction perpendicular to that of the continuous phase (Figure 2b). The FFD (Figure 2c) is characterized by having disperse phase flow asymmetrically pinched by the continuous phase flow [43]. 

Over the past four years, literature on this subject has been enriched with papers focusing on the use of microfluidics for synthesizing polymer nanoparticulate drug delivery systems, and related studies on microfluidic design and process parameters. Synthetic biodegradable polymers, such as polylactide (PLA), polylactide-co-glycolide (PLGA), and PEGylated PLA and PLGA (PLA-PEG and PLGA-PEG) were involved. Baby and coll. investigated five microfluidic devices with different channel widths and heights ranging between 20 to 100 μm and 50 to 200 μm, respectively. The authors demonstrated the potential of using simple 2D devices for the large-scale production of PLGA-PEG NPs with a suitable and controllable size (20–200 nm) and good homogeneity (polydispersity index (PDI) 0.2) [44]. Lim and coll. evaluated the parallelization of 3D hydrodynamic flow focusing (HFF) in a multilayer microfluidic system. They obtained good results enhancing the production of PLGA-PEG NPs without losing reproducibility, size control, and robustness [37]. The aim of study was to produce batches of nanoparticulate drug delivery systems for clinical application. Chiesa and coll. Investigated the loading of a small hydrophilic drug, such as N-acetylcysteine, into PLGA NPs, and compared the traditional bulk mixing with the microfluidic technique in terms of yield of production and drug loading. The authors achieved significant improvement of drug loading and narrower particle size distribution when PLGA NPs were produced by microfluidic technology. Moreover, in vitro drug release was significantly slower for N-acetylcysteine loaded into NPs. The authors attributed this result to the higher fraction of the drug encapsulated into the polymer matrix, whereas N-acetylcysteine is mainly absorbed at the surface of NPs that are produced by bulk mixing [45]. Abalde-Cela and coll. applied the microfluidic technique to the synthesis of branched gold nanoparticles, with high quality results in terms of process reproducibility [46]. Panariello and coll. set up a mathematical model for the synthesis of spherical NPs in a continuous flow microreactor and applied the model to a case study (i.e., the synthesis of silica NPs using the microfluidic technique). They studied the parameters, such as residence time and difference, in liquid diffusivity along the process [47]. As a conclusion of this chapter, it can be stated that the microfluidic technique applied to the synthesis of NPs gives notable advantages in terms of process speed and reproducibility, and drug loading ability.

## 3. Polysaccharide are Commonly Investigated as Nanoparticulate Drug Delivery Systems

Polysaccharides are promising polymers in drug delivery systems. The application of the microfluidic technique to these polymers for the manufacturing of NPs is a topic of increasing interest in the scientific literature [48,49,50,51,52]. These polymers have many advantages over synthetic polymers, such as their natural abundance, generally low cost, easy manipulation, and derivatization due to the presence of several nucleophilic groups in their molecules. Moreover, in most cases, they are biocompatible [53].

The most commonly studied polysaccharides for nanoparticulate drug delivery systems preparation are chitosan, hyaluronic acid, and alginate. All of them are biocompatible, biodegradable hydrophilic polymers, recognized as safe by regulatory agencies, and their use is approved in drug products. Specific polymer properties and structures are summarized here below. All these natural polymers are charged (alginate, hyaluronic acid, chitosan), mainly because of the presence of ionizable groups along the chain. The influence of the charge type and density on the cellular response is not fully understood. However, many negatively charged biomaterials do not induce a strong inflammatory response, in contrast to positively charged polymers that tend to attract inflammatory cells [54]. 

These polysaccharides are obtained from natural sources. They must be purified before use for pharmaceutical and biomedical purposes, in order to eliminate foreign matter that could cause the body immune response after implantation, and to get homogeneous batches. However, the latter point is an issue when working with natural materials.

### 3.1. Chitosan (CS)

**Figure 3:**
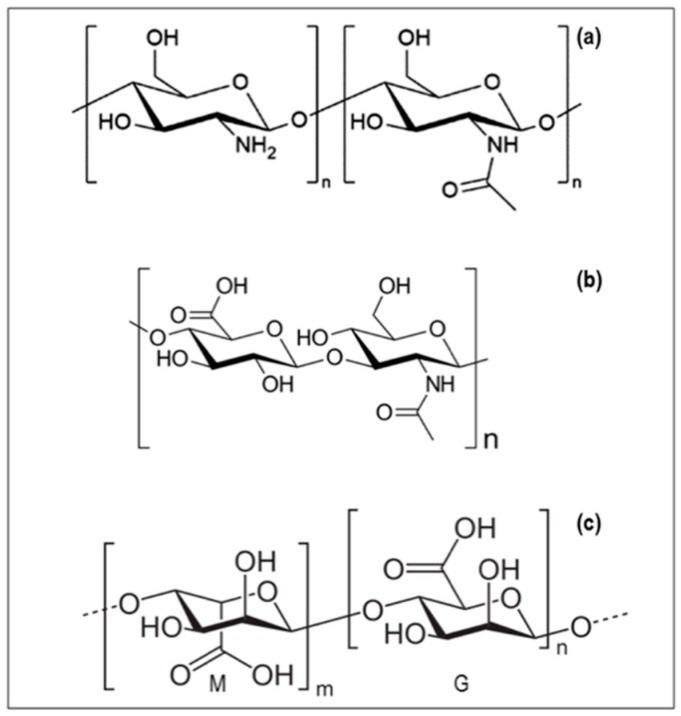
Molecular structures of: (**a**) Chitosan (CS); (**b**) hyaluronic acid (HA); and (**c**) alginic acid (M—mannuronic acid unit, G—guluronic acid unit).

Chitosan (Figure 3a) is a family of polysaccharides characterized by a randomly distributed β-(1→4)-linked D-glucosamine and *N*-acetyl-d-glucosamine monomers, with a wide range of molecular weights (M_W_), deacetylation degree (DD), and *N*-acetylation pattern (PA). The most common source of chitosan is the exoskeleton of marine organisms (e.g., crustacean shells and squids), however they can be also produced by specific mushrooms [55,56]. Chitosan is biodegradable and biocompatible with low immunogenicity, and has interesting biological properties, such as antimicrobial, antioxidant, and antifungal activities. This polymer is used in different sectors, spanning from biomedical to cosmetic and from food to agriculture. One of the most investigated properties of chitosan is its antimicrobial effect [57,58].

The main characteristic of chitosan is the presence of amino groups on its backbone that gives it a positive charge. This charge confers peculiar characteristics to chitosan, such as muco-adhesiveness, antimicrobial activity, and enhanced permeability. The positive charge density of chitosan amino groups depends on their DD and the degree of substitution (DS) of amino groups. DD affects chitosan gene transfection efficiency, and chitosan DNA binding. Therefore, DD is a parameter of utmost importance when designing chitosan-based nanoparticulate drug delivery systems [59]. The antimicrobial effect of chitosan is due to its positively charged amine groups binding to the negatively charged surface of a bacterial wall or plasmatic membrane. This causes a modification in cell permeability, leading to osmotic damage with an efflux of ions and proteins from the cytoplasm to the extracellular space [58,59,60]. Low-molecular-weight chitosan can penetrate bacterial cells’ walls, and they can bind DNA-inhibiting DNA transcription and mRNA synthesis [61,62,63]. High-molecular-weight chitosan can bind negatively-charged components on bacterial cells’ walls, making an impermeable layer that changes cell permeability and blocks transport into the cell [64]. The antimicrobial effect of chitosan is more pronounced in Gram-negative than Gram-positive bacteria [65].

A strategy to obtain high-charge density is to prepare chitosan ammonium quaternary derivatives. In this way, the chitosan backbone achieves a permanent positive charge and the polymer highly interacts with the membranes of bacterial cells.

The mucoadhesion of chitosan, and its cationic derivatives, is recognized and proved to enhance the adsorption of drugs, especially at neutral pHs. *N*-trimethyl chitosan chloride is one of the most studied chitosan derivatives, due to its interaction with negatively charged cell membranes [55]. 

Due to its favorable properties, chitosan has been investigated as a biomaterial in nanomedicine, leading to the formulation of several mixed nanosystems (e.g., NPs, nanocomposites) [58]. 

Chitosan NPs exhibit outstanding biodegradable and biocompatible properties, and for these reasons they are extensively studied as drug carriers. Studies have shown that chitosan NPs can deliver many drugs, including genes, proteins [66,67,68], anticancer chemical drugs [69,70], and antibiotics [71,72]; and via various routes of administration, including oral, nasal, intravenous, and ocular [73,74,75]. CS–TPP NPs have been investigated for the delivery of anticancer and protein drugs by oral administration [76,77,78]. Amidi and coll. [79] investigated the potential of N-trimethyl chitosan (TMC) NPs as a carrier system for the nasal delivery of proteins. 

Moreover, chitosan NPs can be designed with suitable properties such as slow and controlled drug release, improving drug solubility and stability. 

In summary, chitosan NPs exploit the main aims of drug delivery systems (i.e., to enhance drug efficacy and to reduce drug toxicity). Moreover, their small size enables them to cross biological barriers in vivo (such as the blood–brain barrier) and deliver drugs to lesion sites [76]. Basically, the main mechanisms for the preparation of chitosan NPs rely on the formation of polyelectrolyte complexes, commonly referred to as ionotropic gelation. This is an eco-friendly and safe NPs preparation method, based on ionic interactions between the positively charged primary amino groups of chitosan and the negatively charged groups of polyanions, such as sodium tripolyphosphate (TPP) hydroxypropyl methylcellulose phthalate and hyaluronic acid (HA). Over the years the interest in the production and formulation of chitosan NPs has increased dramatically [79,80,81,82].

### 3.2. Hyaluronic Acid (HA)

HA (Hyaluronic acid, hyaluronan) is a mucopolysaccharide belonging to the glycosaminoglycans (GAGs) family, and is composed of disaccharide units of d-glucuronic acid and N-acetyl-d-glucosamine (Figure 3b). Other commonly known compounds, such as chondroitin sulfate, keratan sulfate I and II, heparin, heparan sulfate, and dermatan sulfate are included in the class of mucopolysaccharides [83]. 

HA is a biopolymer that is naturally occurring in the human body, namely in skin, connective tissue, synovial fluid, eye vitreous body, and intervertebral discs. It is synthesized by hyaluronan synthetases and enzymatically degraded by hyaluronidases. Moreover, HA is the only mucopolysaccharides whose synthesis does not happen in the Golgi apparatus. It provides cellular support, making a hydrophilic matrix that enables cell–cell adhesion, cell migration, as well as cell growth and differentiation. It exploits several functions in the human body, such as the control of tissue hydration and water transport, and the maintenance of the viscoelasticity of liquid connective tissues (e.g., joint synovial fluid and eye vitreous fluid). HA is involved in the supramolecular synthesis of proteoglycans in the extracellular matrix, and in various, mediated receptor roles, such as in tumor development, metastasis, inflammation, cell mitosis, migration, and detachment, and it is accepted to have a significant role in wound and tissue repair processes. The last role, mainly ascribed to high molecular weight (Mw) HA, is due to HA interaction with oxygen-derived free radicals (ODFR), resulting in a significant protective activity for chondrocytes against ODFR action [72,84,85,86,87,88]. 

HA can be divided into groups by Mw: High Mw HA (HMw HA) >30 kDa and low Mw HA (LMw HA) <30 kDa. HMw HA is recognized to be antiangiogenic and non-immunogenic, whereas LMw HA is regarded as inflammatory, immuno-stimulatory, and angiogenic. Nevertheless, not all literature reports are consistent, revealing that HA has more complex behaviors and mechanisms [85,89,90].

HA ability in maintaining tissues elasticity and hydration, and in the entrapment or permeation of small and large molecules, derives from its viscoelasticity and high hydrophilicity [91]. These are two important interdependent properties that should always be evaluated.

The high biocompatibility and lack of immunogenicity of Low Mw HA favored its use in numerous clinical applications. For example, in orthopedics it is widely used as a joint fluid supplement in osteoarthritis and rheumatoid arthritis, which is also due to the natural occurrence of HA in the synovial fluid, joint capsule, and articular cartilage. HA has multiple applications in ophthalmology, both in the conservative and in operational aspects. It is frequently used as the “lubricant” component, thanks to its viscoelastic properties, often being the main ingredient in artificial tear formulations, used in the relief of dry eyes. It reduces irritation, moisturizes the eye, and replenishes the deficiencies of sodium hyaluronate in tear film. Sodium hyaluronate is currently the most commonly used cosmetic filler, with the goal to fill and plump up the extracellular tissue space. In liquid formulations, it is injected to fill small, superficial wrinkles, giving the skin elasticity and flexibility, and cross-linked preparations are used for the correction of facial contours and for modelling breasts in women, thorax in men, and buttocks in both sexes [91].

HA can be complexed with chitosan in order to coat chitosan NPs and to improve chitosan–TPP complexation [92,93,94]. Recent published studies reported better toxicological profile, muco-adhesiveness, and cell adhesion properties when chitosan NPs were labelled with HA. All these properties indicated an improved cell targeting ability [95].

HA based NPs are studied in oncology drug delivery, focusing on selective targeting of HA towards the CD44 and CD168 membrane receptors. The presence of HA on the surface of NPs endows the carriers with active targeting towards these HA receptors, which are highly expressed on various tumors such as squamous cell carcinoma, ovarian, colon, stomach, glioma, and many types of leukaemia, lymphoma, and myeloma. The drugs can be encapsulated in the HA-based NPs, or they can be directly linked to HA. Ultimately, HA itself can be conjugated to NPs of different polymer matrices (e.g., chitosan), or it can be used as the coating of nanosized objects of inorganic material (e.g., gold) [95,96]. Because the topic is of particular interest in drug delivery, here below an in-depth analysis is introduced.

#### 3.2.1. HA-Binding CD44 Receptor

**Figure 4:**
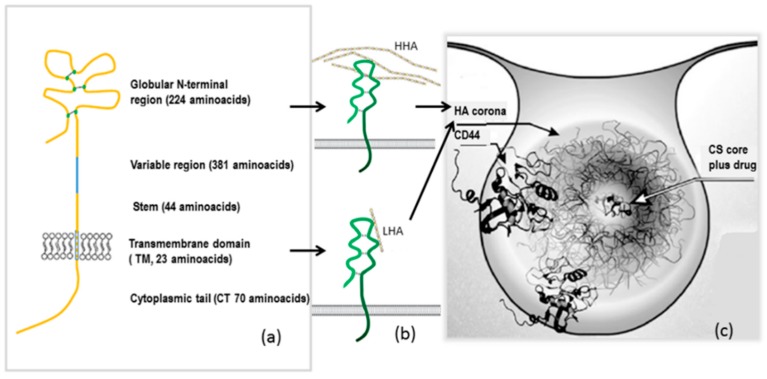
(**a**) CD44 structure; (**b**) CD44–HA interaction with high molecular weight HA (HMw HA) or low molecular weight HA (LMw HA); and (**c**) endocytosis of HA-coated chitosan (CS) nanoparticles (NPs), mediated by interaction with the CD44 receptor, adapted with permission from [83,96,97].

CD44 is a stem-like cell receptor (also known as H-CAM, Hermes Ag, and human phagocytic glycoprotein-1). The CD44 is a family of transmembrane glycoproteins that function in extracellular adhesion and signal transduction. CD44 gene is on chromosome 11 in humans, and encodes for a protein with a cytoplasmic domain (70 amino acids), a transmembrane domain (23 amino acids), and an extracellular domain. This last domain includes a stem region (proximal to cell membrane; 44 amino acids), a variable region (up to 381 amino acids), and a globular N-terminal region (224 amino acids), where ligand binding occurs (Figure 4a) [84,96].

The CD44 gene is composed of 20 exons. Ten exons (known as “constant” exons) are expressed in all isoforms. The ten central exons, known as “variant” exons, are excised or included in various combinations by alternative splicing in the membrane-proximal stem region. They account for the heterogeneity of this protein family. The last two exons encoding in the CD44 cytoplasmic domain are also subjected to alternative splicing. CD44’s smallest isoform (CD44s), which lacks all variant exons in the extracellular domain, is ubiquitously expressed, whereas the expression of variant isoforms is confined to only a few tissues under specific conditions. The structural diversity of CD44 further amplifies by extensive and often isoform-specific post-translational modifications, including N- and O-linked glycosylation, phosphorylation, and glycosaminoglycan attachment. The first five exons on CD44 encode an amino-terminal globular protein domain, which contains ligand-binding receptors for HA. The introduction of new exons modulates HA-binding affinity by inducing conformational changes or allowing CD44 isoforms to have new functions.

CD44 expression is associated with stem cells. Therefore, CD44 is a common stem cell biomarker, and a marker for cancer stem cells (CSCs), which are otherwise known as cancer initiating cells. CD44 binding regulates CSCs survival, self-renewal, maintenance, and chemo resistance. For this reason, CD44 is critical for disseminated cancer cells to adapt to new environments.

CD44 or CD44-like receptors are significantly over-expressed in different solid tumors, including lung cancer, pancreatic cancer, and breast cancer, thus, tremendous efforts have been focused on studying the diagnostic and prognostic value of CD44, and particularly CD44v isoforms, in cancer. High expression levels of CD44 are associated with drug resistance during cancer metastasis and are a marker of unfavorable prognosis in some cancers. Therefore, targeting CD44 is considered a valuable therapeutic design. However, the targeting strategies should consider that CD44s exists on many normal cells and their expression have not been extensively studied yet [83,97,98,99].

Hyaluronic acid has high affinity for the CD44 receptor. The CD44 receptor has been demonstrated to interact with HA at the N-terminus of its extracellular domain, serving as major cell surface receptor for HA. High molecular weight HA (HMw HA) in ECM is degraded by hyaluronidases into low molecular weight (LMw HA) fragments that can still bind to CD44. Interestingly, HMw HA and LMw HA often exert opposite effects on several physiological and pathological processes. It has been suggested that the different biological effects reported for HMW HA and LMW HA should be mediated by CD44. HMW HA promotes cell invasion in different types of tumors, such as breast, brain, pancreatic, and lung tumors. The high affinity of HMW HA (as opposed to the low affinity of LMW HA) is probably a result of multivalent binding, because HMW HA contains thousands of binding sites. Moreover, HMW HA has been demonstrated to promote the long-term circulation and increased stability of NPs after injection, because it reduces protein adsorption (corona) to NPs following opsonization. For all these reasons, CD44 and HA interaction can be exploited as a potential target for cancer therapy. The inherent ability of HA to target cell membrane receptors is studied and exploited in drug delivery through the formulation of HA nanoparticles loaded with anticancer drugs. Literature reports many studies on HA-based NP drug carriers for the targeting of tumor tissues. It is well known, from the literature, that nanocarriers passively accumulate in tumor areas due to their ability to take advantage of the enhanced permeability and retention (EPR) effect. The targeting of tumor cells can be selectivity enhanced, through active targeting, by conjugating an active moiety on the surface of NPs. HA-based NPs bind to CD44 and are entrapped, to a greater extent, into cells that over-express CD44 receptors, delivering the active compound. This mechanism is particularly active in CD44-positive and drug-resistant cancer stem cells, and it can be achieved either via drug conjugation to HA or by entrapping the drug in HA or HA-modified NPs or micelles. The objective is to achieve active-targeting drug delivery systems, creating a selective cancer therapy with minimal systemic toxicity. The bond between HA and the CD44 receptor is influenced by cell type and CD44 isoforms, so sometimes the interaction can be minimal or an activation process can be required [84,89,98]. In conclusion, suitably studied HA binding with CD44 permits a receptor-mediated endocytosis, which facilitates drug transport inside tumor cells and contributes to improve drug cytotoxicity at the site of action (Figure 4b) [84,100].

#### 3.2.2. HA and CS Nanoparticles

HA is often combined with CS when making NPs with some promising advantages. Here below, some examples from the literature involving studies on the use of HA and CS NPs as drug delivery systems for different purposes, and their advantages, are discussed.

When CS is the main component of the NPs (CS NPs), these systems possess a cationic surface, which significantly reduces their circulation time and bioavailability upon exposure to a biological environment. However, when these NPs are decorated with anionic polysaccharides, such as hyaluronic acid (HA), both protein adsorption and the rate of macrophage uptake decrease. As reported and discussed above (see Section 3.2.1), the presence of HA on the surface of CS NPs (HA-CS NPs) allows for targeted delivery to cells bearing CD44 receptors [101]. Another example in the literature exploited HA conjugation to docetaxel (DTX) to obtain a water-soluble molecule (i.e., HA-DTX conjugate), overcoming the low-water solubility of DTX. Moreover, HA conjugation to DTX could improve drug targeting to cells overexpressing CD44. The authors synthesized the water-soluble conjugated hyaluronic acid-docetaxel (HA-DTX), and afterwards they prepared chitosan coated HA-DTX NPs by way of interaction of the anionic structure of HA-DTX and the cationic chitosan. The authors claimed HA could cause heparin-induced thrombocytopenia as justification of the chitosan coating; they demonstrated the chitosan coating had a positive zeta potential, which in turns promoted NPs stability. The paper is interesting; however, the authors did not explain if the chitosan coating decreased HA targeting towards CD44 [102].

Other examples of studies involving HA and CS NPs in drug delivery involved nucleic acid-based therapeutics. In recent years, nucleic acid-based therapeutics are envisioned to play a significant role in the next generation of treatments for a variety of diseases, such as cancer. The demonstration that RNA interference was mediated by small-interfering RNA (siRNA) that operated in mammalian cells opened up new possibilities to develop highly-specific RNA-based gene-silencing therapeutics [103]. Several clinical trials, using small interfering RNA (siRNA) as a therapeutic molecule, demonstrated RNA interference (RNAi) as a promising option to treat diseases. However, diverse physiological limitations related to siRNA size and negative charge hinder its trafficking within the blood stream and reduces siRNA transfection. To overcome these drawbacks, siRNA vector-assisted delivery strategies and polymer conjugation involving HA and CS NPs are studied [104,105,106].

Al-Qadi and coll. have exploited the addition of HA to CS-based NPs. Indeed, inclusion of HA provided a variety of gene delivery systems with improved, synergistic properties, through different mechanisms. HA proved to reduce non-specific interactions of these systems with serum proteins, and at the same time it improved their cell internalization. Therefore, HA, being included in lysosomal vesicular compartments, rapidly accumulated in the perinuclear region and cell nuclei, favoring gene expression. Moreover, HA was believed to act as a transcriptional activator, probably by loosening the tight binding between the gene and carrier in question [103].

Other examples of studies on HA and CS NPs involve growth factor (GF)-based therapies in regenerative medicine. Despite the unquestionable interest of GF-based therapies, a critical limitation of these molecules is their extremely fast degradation in physiological conditions. This problem does not permit GFs to exercise a continuous, long-lasting effect. An additional disadvantage of GFs injected as solutions is their indiscriminate distribution throughout the body that may lead to undesirable side-effects. To overcome these limitations, several strategies based on drug delivery technologies have been proposed. Chitosan (CS) and hyaluronic acid (HA) NPs have superior tissue regenerative properties. Beyond its selective interaction with CD44 receptor, HA is a natural component of human ECM, and chitosan has properties such as penetration enhancement, antibacterial activity, etc. From this background, Parajó and coll. propose HA and CS as carriers for proangiogenic growth factors, namely vascular endothelial growth factor (VEGF) and platelet derived growth factor (PDGF-BB). They prepared HA and CS NPs by ionotropic gelation, adding stabilizing agents such as bovine serum albumin and heparin sodium. They achieved efficient entrapment of both growth factors and prolonged the release up to one week for PDGF-BB, while VEGF was released in 24 h. The authors hypothesized that the NPs could be a profitable drug delivery system for PDGF-BB in treating ischemia and in tissue engineering. They would expect that the combination of a continuous stimulus from the controlled release of GF and the potential biological activity exerted by HA could lead to a significantly enhanced therapeutic effect, as compared to the GF in solution [107]. Recently, HA and CS-TPP NPs were investigated as carriers for everolimus, a poorly water-soluble drug and a key moiety in the prevention of allograft organ rejection. The rationale of this work was the selective delivery of everolimus into lung allograft-derived mesenchymal cells, in order to treat bronchiolitis obliterans syndrome (BOS), which is the main cause of lung rejection. The authors set up, through a design of experiment approach, a one-step ionotropic gelation method to prepare HA-decorated CS-based NPs with a high outer HA deposition on the surface of NPs. They reached a satisfactory drug entrapment, which released up to 24 h. The efficacy of the NPs and their targetability to CD44 was tested on bronchial mesenchymal cells from BOS patients with positive results. The authors concluded that the combination of safe polymers, such as CS and HA, and the ability to selectively hit diseased cells could open up new perspectives for a more effective pulmonary therapy for BOS [108].

Another example of HA and CS-TPP NPs found in the literature involves ocular drug delivery. The corneal epithelial layer, aqueous-vitreous humors, blood–aqueous humor barrier, and the blood–retinal barrier limits the entry of hydrophilic or hydrophobic moieties into eyes, eventually with very low bioavailability of drugs. Due to these protective mechanisms, drug delivery through tight junctions of corneal epithelium is very difficult. Hence, the development of suitable drug delivery systems is needed to attain effective drug concentrations into eyes for a prolonged time after dose instillation. Mucoadhesive polymers and mucoadhesive polymer-based nano-formulations are used and studied to increase the bioavailability of drugs after ocular administration [109,110]. Starting from these, background studies on NPs made of hyaluronic acid (HA) and chitosan (CS), and intended for the delivery of complex macromolecules to the ocular surface, have been performed. HA-coated CS-NPs were selected due to HA’s ability to improve cellular targeting by interacting with CD44 receptors, which was expressed on human corneal and conjunctival epithelia cells, and also used to promote the regeneration of corneal and conjunctival epithelial cells [109,111,112,113]. Kalam and coll. evaluated the potential of a CS-TPP-based nanocarrier coated with HA, designed for the targeted and intracellular delivery of dexamethasone sodium phosphate (DEX) into the eyes. The goal was to improve DEX precorneal retention and corneal permeability. The paper is an example of an accurate study on NPs’ in vitro characterization and stability. The authors used a two-step preparation process: They obtained CS NPs loaded with DEX by ionotropic gelation; afterwards they performed the coating with HA, with the goal to improve the mucoadhesion of the NPs. They obtained NPs with a highly negative surface charge, which was predictive of good physical stability towards potential particle aggregation. The particle size of the CS NPs was about 300 nm and increased up to 400 nm after HA coating. Polydispersity index was about 0.1 before lyophilization and significantly increased after NPs lyophilization (up to 0.4), but the NPs were stable in size and zeta potential in a three month storage study. The in vitro release kinetic was estimated to follow a Fickian diffusion kinetic [99]. Wadhwa and coll. prepared HA-modified CS NPs (CS-HA-NPs) loaded with timolol (TM) and dorzolamide hydrochloride (DH), which are drugs typically administered in the treatment of glaucoma. The two polymers were selected for their mucoadhesive properties and their potential to reduce irritation after local administration into eyes. The CS-HA nanoparticulate drug delivery systems were characterized in vitro, tested on rabbits’ eyes, and demonstrated to be potentially advantageous in both reducing inflammation caused by local drug administration and in prolonging the drug effect. The authors highlighted the importance of drug carrier mucoadhesion, more than selective cell targeting, to improve glaucoma therapy [113].

Other authors exploited HA and CS mucoadhesion to improve the delivery of macromolecules, such as heparin. For example, Oyarzun-Ampuero et al. proposed nanocarriers made of chitosan and hyaluronic acid for pulmonary delivery of heparin in the treatment of asthma. The NPs were obtained by ionotropic gelation by CS and HA, and TPP was used as the crosslinking agent. They showed, in ex vivo experiments, that heparin-loaded CS-HA NPs prevented histamine release in rat mast cells. This positive result led the authors to assume that the heparin-loaded CS-HA NPs were a potential effective delivery system [92].

## 4. Synthesis of Polysaccharide NPs by Microfluidic Devices

The chapter focuses on some examples of literature on the synthesis of polysaccharide NPs using microfluidic devices. The authors do not intend to be exhaustive, but rather to discuss significant examples in order to highlight the properties and advantages of microfluidic devices in the synthesis of polysaccharide NPs, namely for drug delivery applications. Studies on the preparation of polysaccharide NPs using the microfluidic technique are quite recent and not so abundant.

Majedi and coll. [49] employed a T-shaped PDMS microfluidic device with three inlets and one outlet for the formation of NPs. Using this method, the authors prepared chitosan (CS) NPs by way of ionic gelation. The aim of the proposed NPs was to to obtain proton exchange membrane fuel cells with improved performances, and therefore the authors selected adenosine triphosphate (ATP) as the crosslinker and anhydrous proton conductor via adenine group. They investigated the process using sodium fluorescein as the marker to highlight the flow stability in the microchannel and, eventually, the flow-focusing ability. They demonstrated that the extent of fluorescein stream focusing can be controlled by the flow rate ratio. In this work, the mixing time was controlled in the millisecond range, which is typical of the microfluidic technique. NPs of about 100 nm, smaller in size and more homogeneous than the particles prepared by slow (bulk) mixing, were obtained with low polydispersity index (PDI 0.2). This NP size was particularly suitable to the authors purpose, because particles with diameters larger than 100 nm cannot enter the ionic nanochannels of Nafion-based nanocomposite membranes. 

Indeed, Majedi and coll. studies explored microfluidic process application in the fabrication of chitosan NPs for drug delivery [48,114]. The authors used the same technique explained above (i.e., T-shape PDMS microfluidic device with three inlets and one outlet) to prepare hydrophobically-modified chitosan (HMCS) NPs loaded with an anticancer drug, such as Paclitaxel. The interesting point and novelty reported in these papers was the pH triggered self-assembly mechanism that was exploited to get NPs with. HMCS molecules self-assemble at pH 7.4. The authors kept mixing time in the millisecond range, from 2.5 to 75 ms, by changing the flow ratio of the chitosan solution (pH 5.5.) to water (pH 9) streams, from 0.03 to 0.2, up to achieving the self-assemble pH. They obtained kinetically-stable NPs with a positive surface charge of almost three times higher than the surface charge of NPs obtained by bulk mixing. The NPs had a small size, ranging between 75 and 122 nm. Moreover, the fabrication process was solvent free, and the acidic pH prevented paclitaxel aggregation during the nanoparticle preparation process. The in vitro drug release rate from the NPs in the first 24 h was slower if compared with the release rate from NPs obtained via bulk mixing (80%), but nevertheless was always quite high (40%), depending on the amount of drug at the NPs surface. Typically, the slower mixing time corresponds to the higher burst effect because the highly hydrophobic drug tends to accumulate at the surface of NPs. Moreover, the paclitaxel release rate was triggered by the environmental pH, and it is one order of magnitude faster at pH 5.5 (this pH is common in a tumor site). This example shows the superiority of microfluidic mixing in comparison with bulk mixing. Rapid mixing, precise tunability, and reproducibility, independent of user talents, are difficult to obtain through conventional methods. Microfluidics has made these parameters more precisely obtainable by introducing precise set up of flow ratio, mixing time, and chip characteristics. In particular, the ability to set flow ratio in the millisecond range is typical of this technique and permits the obtainment of superior NPs, as long as particle size and drug loading is concerned. This example shows the superiority of the properties of particles obtained by the microfluidic mixing technique, in comparison with the particles obtained through bulk mixing.

Bicudo and coll. [115] used a microchannel flow-focusing device to study the production of HA NPs crosslinked with adipic hydrazide (ADH) and chloride carbodiimide (EDCl). The process is a continuous nanoprecipitation at the water–organic solvent interface, and the study is focused on evaluating the process parameters of the innovative process. The authors analyzed the influence of the type of organic solvent used, non-solvent flow rate, and the HA concentration on the properties of HA NPs. The investigation results showed that the affinity between water and organic solvent controlled the mean diameter of the NPs through water diffusion and nanoprecipitation rates. Narrower polydispersity was obtained when non-solvent had intermediate affinity for water. In addition, lower HA concentrations and higher isopropyl alcohol flow rates produced smaller particles, as the process was convection controlled. Moderately stable NPs were obtained irrespective of the organic solvent, its flow rate, or HA concentration. The process was confirmed to be simple, reproducible, and fast. The authors concluded that this procedure was promising for producing HA NPs that were free of oil and surfactants, which is important for medical, pharmaceutical, and cosmetic applications.

**Figure 5:**
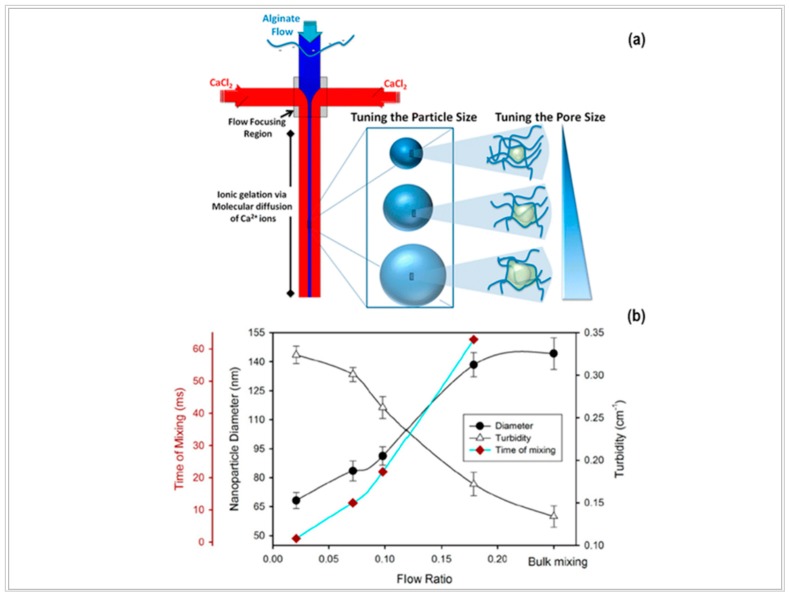
(**a**) Schematic representation of the microfluidic-assisted approach for the synthesis of alginate NPs, and (**b**) the correlation of the size and compactness of NPs with the process (adapted with permission from [116]).

Bazban-Shotorbani and coll. employed a cross-junction poly(dimethylsiloxane) (PDMS) microfluidic device, with three inlets and one outlet, to synthesize alginate nanogels crosslinked with CaCl_2_, and with tunable size and porosity, Figure 5a [116]. The authors studied the influence of mixing time on the average size and pore size (compactness) of NPs, which were fundamental in determining the drug release from NPs. They demonstrated a correlation on a mathematical basis. The paper is interesting because it gives a correlation between the properties and synthesis parameters of NPs, and it shows that microfluidics provides a controlled and tunable platform. Briefly, starting from Equation (6), the theoretical mixing time (τ_mix_) was estimated according to Equation (7):

τ_mix_ ≈ (w_f_^2^)/4D ≈ (w^2^)/9D *1/ (1+ 1/FR)^2^ (7)

where D is the diffusivity of the calcium ions, w_f_ is the width of the focused stream, w is the channel width, and FR is the flow rate ratio of the polymeric stream to the total water flow rate. The authors explored a wide τ_mix_ in the range 2–65 ms. The size of NPs was measured by dynamic light scattering (DLS) in gastric- and intestinal-simulated fluid. The results showed that the size of NPs increased as FR or polymer concentration increased (Figure 5b). The authors explained that the effect was due to the higher amounts of alginates available to react with Ca^2+^ ions (whose concentration was always in excess). Moreover, the authors found out that the polydispersity index (PDI) of NPs prepared by the microfluidic device was always smaller than the PDI of NPs prepared by bulk mixing. They suggested that the narrower PDI of NPs prepared by the microfluidic device was due to the shorter residence time in the flow stream and the absence of turbulent and convective motions. For this reason, a strict correlation between polymer solution concentration and the size of NPs took place. Eventually, on the basis of Mie theory, the authors calculated a correlation among the compactness, polymer solution concentration, and flow rate of NPs. They concluded that the compactness of NPs decreased by increasing the focus stream flow rate.

Kim and coll. [117] introduced the generation of alginate-based NPs through polyelectrolyte complexation in a microfluidic device. The polyelectrolyte complexation was carried out between anionic Ca-alginate pre-gel (of extremely low alginate concentration, 0.05 *w*/*w* %) and cationic Poly-L-lysine (PLL) solution (whose concentrations tested were among 0.25 and 1.50 *w*/*w* %). Ca-alginate pre-gel flowed co-currently into the PLL solution at a controlled rate. A purposefully modified microfluidic device was used. Results showed that the size of alginate NPs was controlled from 380 nm to 520 nm by changing the flow rates of the Ca-alginate pre-gel and PLL solutions. PLL concentrations interacted with alginate molecules, thus affecting the size of NPs; high PLL concentrations decreased the charge inside the complex, enhancing polymer molecule attraction and the stability of the NPs. As a result, the NPs were best stabilized at the critical PLL concentration of 1 *w*/*w* %; higher PLL concentrations led to the development of irregular shaped NPs. This PLL concentration was higher with respect to that used to prepare the same NPs in the traditional bulk mixing process. The obtained NPs showed enhanced stability and size uniformity compared with those obtained using the conventional bulk mixing method. The method developed in this study was processed entirely in an aqueous mild environment without organic solvent, and exploits low cost biocompatible polymers. The authors concluded that the generated alginate NPs had great potential to be used in various bioapplications, such as drug delivery, food processing, and enzyme immobilization.

Dong and coll. [118] investigated microfluidics to prepare self-assembled amorphous drug-polysaccharide nanosized complexes (nanoplexes), and they compared the nanoplexes with those obtained by bulk mixing. The authors used perphenazine (PPZ) as the model of a poorly soluble, cationic drug, and dextran sulfate (DXT) as the anionic polysaccharide model. Comparison between microfluidics and bulk mixing showed that the two synthesis platforms shared similar dependence on pH and charge ratio, with similar optimal values for both. Residence time and tube diameter had relatively insignificant impacts on the nanoplex preparation. PPZ–DXT nanoplexes, prepared at the optimal conditions of each platform, had similar size (70–90 nm), zeta potential (50 mV), preparation efficiencies (30% yield), drug dissolution rate, and physical stability during storage. However, significant differences were found in the drug payloads: The nanoplex prepared by bulk mixing exhibited a lower payload with respect to nanoplexes prepared using the microfluidic technique (i.e., 65% vs. 85%). However, the lower payload led to slightly superior supersaturation generation. Nevertheless, microfluidics demonstrated superior production consistency and scalability. This example shows how a preparation technique cannot be defined as good or not as an absolute statement, but it should always be evaluated in its context, which means with respect to the specific drug and polymer interaction. For example, in the reported case the superiority of the microfluidic technique was recognized only for its production consistency.

Li and coll. [119] proposed an original approach to prepare cyclodextrin-based NPs (CD-NPs), whereby dextran (Dex) is grafted with benzophenone (Bz) moieties (Dex-Bz). It is known that Bz forms inclusion complexes with poly-cyclodextrin, and its incorporation into cyclodextrin is a strategy used to improve the water solubility of the poorly soluble compound. The Dex-Bz conjugation was performed in order to improve Bz loading into poly-cyclodextrin, and microfluidics was investigated as an alternative method to produce NPs that were starting from poly-cyclodextrin and Dex-Bz. The two polymers spontaneously connected in water, producing NPs due to the strong interaction of CD with Bz hydrophobic moieties. The microfluidic technique exploited the NanoAssemblr^TM^ Benchtop instrument (Precision NanoSystems, Ltd., Vancouver, BC, Canada), and NPs were prepared in a completely aqueous environment. The formation of NPs was deeply investigated as far as particle size and particle size stability were concerned. In situ size analysis using the VASCO-flex particle size analyzer was carried out during the preparation of NPs, giving consistent results compared to DLS (dynamic light scattering) analysis. The resultant particle sizes were about 100 nm, with narrow size distribution, and the resultant NPs resulted were stable upon storage at room temperature for six days, and upon 1:100 dilution. Nanosight analysis (Nanosight LM10 Instrument, Malvern Instrument Ltd., Malvern, UK) was used to evaluate the size stability of NPs upon storage at room temperature and dilution. The apparatus permitted the ability to visualize single particles in a concentration range between 10^6^ and 10^9^, and to get size distribution and concentration results at the same time. The microfluidic device that was set up and used to produce NPs showed to be advantageous in terms of yield of production and the uniformity of the particle sizes of NPs, when compared to the traditional formation of NPs using pipette dripping. The paper is interesting because the preparation process was combined with a detailed particle size characterization carried out on NPs.

## 5. Conclusions

The application of the microfluidic technique for the synthesis of polysaccharide NPs is currently a topic of interest in the subject of drug delivery. Although a lot of research papers have been already published on the use of the microfluidic technique for the preparation of NPs based on synthetic polymers, the synthesis of polysaccharide NPs using the microfluidic technique has not been so widely explored yet.

After critically evaluating the reported research papers, it can be said that all authors agree that microfluidic devices are superior to other techniques in allowing better control over the preparation procedure of polysaccharide NPs. Thus, the process is more reproducible than the bulk mixing technique.

The technique permits: (i) The efficient and controllable mixing under continuous flow conditions, resulting in a homogeneous reaction environment due to intimate solutions mixing in a narrow environment; (ii) the improved and efficient temperature control and heat transfer; (iii) the temporal reaction control by adding reagent at precise time intervals during the reaction process; (iv) the fast process time, controlled in the millisecond range; (v) the control over the characteristics of NPs by controlling the process kinetics; (vi) the high throughput screening of various formulations by on-line variation of the process parameters; and (vii) the possible scale-up of the process by increasing the number of the microreactors. The resulting products are NPs with superior properties in terms of particle size, particle size distribution, and drug loading.

Moreover, microfluidic devices can be purposefully studied and modified. They can also be integrated with post-synthesis processes and measurement systems on a single technology platform in order to monitor, in situ, the progress of NP formation through residence time-based resolution.

The microfluidic technique lends itself, particularly, to industrial size scale-up, because of its high reproducibility and process yields. Future expected outcomes are: The application of this technique to already set up nanosized products, with the revision of already set up preparation processes of NPs. This could then lead to NP drug delivery systems with enhanced therapeutic efficacy and a lower needed dose. However, it should be reminded that the preparation process must always be studied and adapted with respect to the specific drug and polymer to be processed, because it should never be taken for granted that the microfluidic technique is always the best one.

The high costs of setting up the microfluidic devices for industrial scale-up, that means GMP certified, could be a constraint in transferring this technique to the industrial scale. As far as the pharmaceutical area is concerned, the microfluidic industrial set up could be applied to drug products with high values, such as nanosized drug delivery systems involving monoclonal antibodies, or repurposing old drugs with relevant advantages (e.g., cytotoxic drugs such as doxorubicin, methotrexate, etc.).
